# Early Acute Kidney Injury and Its Association With Survival in Patients With Metastatic Non‐Small‐Cell Lung Cancer Treated With Front‐Line Immunotherapy‐Based Therapies

**DOI:** 10.1002/cam4.71058

**Published:** 2025-07-28

**Authors:** Mingjia Li, Po‐Lan Su, Songzhu Zhao, Grace Cheng, Nicholas Jones, Kenneth Chian, Natalie Johns, Adam Khorasanchi, Adeoluwa Adeluola, Annastasia Petouhoff, Daniel J. Spakowicz, Lai Wei, Xiaoying Wang, Christopher Coss, Mitch A. Phelps, Lingbin Meng, Yuanquan Yang, Alexa Meara, Mohamed I. Elsaid, Jinesh Gheeya, Logan Roof, Asrar Alahmadi, Regan M. Memmott, Jacob Kaufman, Kai He, Carolyn J. Presley, Peter G. Shields, David P. Carbone, Gregory A. Otterson, Jason Prosek, Dwight H. Owen

**Affiliations:** ^1^ Division of Medical Oncology, Department of Internal Medicine The Ohio State University, Columbus USA; ^2^ Department of Biomedical Informatics, Center for Biostatistics The Ohio State University Columbus USA; ^3^ Division of Hospital Medicine, Department of Internal Medicine The Ohio State University Columbus USA; ^4^ College of Medicine The Ohio State University Columbus USA; ^5^ College of Pharmacy The Ohio State University Columbus USA; ^6^ Pelotonia Institute for Immuno‐Oncology The Ohio State University Columbus USA; ^7^ Division of Nephrology, Department of Internal Medicine The Ohio State University Columbus USA

**Keywords:** acute renal failure, immune checkpoint inhibitors, immunotherapy, non‐small‐cell lung cancer, pembrolizumab, survival

## Abstract

**Introduction:**

Immune checkpoint inhibitors (ICIs) have revolutionized metastatic NSCLC treatment. Acute kidney injury (AKI) is a common complication of ICI‐based therapies, alone or with chemotherapy. This study investigates the association between early AKI and 12‐month survival among patients with metastatic NSCLC receiving front‐line ICI‐based treatment.

**Methods:**

This retrospective study included metastatic NSCLC patients who received ICI‐based therapy (2017–2022). Early AKI was defined as a creatinine increase ≥ 1.5 times baseline within 21 days of the fourth cycle or last cycle if fewer than four were given. Clinical characteristics were compared using t‐tests and chi‐squared tests. Survival differences were assessed by Kaplan–Meier and log‐rank tests, with Cox models evaluating the association between AKI and 12‐month survival.

**Results:**

Of the 310 patients, AKI occurred in 38 patients (12.6%), with 8 patients (2.3%) missing follow‐up creatinine data. The hazard ratio (HR) for death within 12 months for patients who developed early AKI was 1.733 (95% CI 1.060–2.835, *p* = 0.026). The highest rate of early AKI was seen in patients receiving pemetrexed, pembrolizumab, and carboplatin (16.7%), compared to 11.1% for pembrolizumab monotherapy and 4.5% for pembrolizumab with paclitaxel and carboplatin. Although patients who recovered renal function were more likely to continue immunotherapy, 12‐month survival rates did not significantly differ (52.2% vs. 46.7%).

**Conclusions:**

Early AKI during pembrolizumab‐based treatment in metastatic NSCLC patients was associated with reduced 12‐month survival. These findings highlight the need for close monitoring and preventive strategies to manage AKI in this population.

## Graphical abstract

Early acute kidney injury (AKI) in metastatic NSCLC patients treated with pembrolizumab‐based regimens was associated with reduced survival within the first 12 months of treatment, with the highest AKI rates observed in patients receiving combination regimens with pemetrexed and carboplatin. This underscores the critical need for effective monitoring and preventive strategies to manage AKI and for studies to evaluate the etiology and biology of the association between AKI and cancer outcomes.

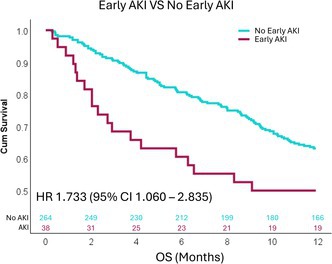

## 1 Introduction

Immune checkpoint inhibitors (ICIs) have transformed the treatment landscape for metastatic non‐small cell lung cancer (NSCLC) lacking actionable mutations [1, 2, 3]. Administered either as monotherapy or in combination with chemotherapy, ICI‐based treatments have demonstrated notable survival benefits in this patient population [4, 5, 6, 7, 8, 9]. However, their use is often accompanied by toxicities including acute renal injury (AKI) [10, 11, 12, 13, 14, 15, 16]. These renal dysfunctions can complicate cancer treatment and significantly influence outcomes [17, 18]. There is a pressing need to better understand the risk, mechanisms, and outcomes of treatment‐associated renal injury to develop more effective preventive strategies.

AKI from ICIs can manifest through mechanisms such as T‐cell‐mediated damage to the kidney resulting in acute interstitial nephritis [19, 20]. In particular, ICIs in the forms of monoclonal antibodies (mABs) targeting Programmed Cell Death 1 (PD‐1), Programmed Cell Death Ligand 1 (PD‐L1), or Cytotoxic T‐Lymphocyte–Associated Antigen 4 (CTLA‐4) can trigger immune dysregulation that leads to inflammatory infiltrate within the renal parenchyma [21]. This injury can range from mild renal dysfunction to severe acute kidney injury leading to permanent renal failure, underscoring the need for early recognition and appropriate management in patients receiving ICI‐based treatment [22, 23].

Chemotherapy is frequently combined with ICIs, particularly in patients with metastatic NSCLC with low and negative PD‐L1 expression [5, 6, 7, 9]. Commonly used chemotherapy agents, such as pemetrexed, paclitaxel, and carboplatin, are known to be directly nephrotoxic [13, 14, 15]. Additional indirect renal damage may result from factors such as dehydration caused by chemotherapy‐induced gastrointestinal side effects, sepsis resulting from immunosuppression, or alterations in the gut microbiomes that increase the production of nephrotoxins like indoxyl sulfate [24, 25]. These complications can further disrupt treatment schedules and compromise therapeutic efficacy [15, 26].

In the early phase of NSCLC treatment, AKI is particularly critical, as it can interrupt life‐prolonging therapies and have a potentially long‐lasting influence on the outcome [27, 28]. By examining the association between early AKI and survival, this study aimed to understand the importance of renal dysfunction in cancer prognosis and emphasizes the need for further research into preventive renal strategies to improve patient outcomes.

## 2 Methods

This retrospective study was conducted at The Ohio State University and included consecutive patients with metastatic NSCLC who received pembrolizumab‐based first‐line therapy between 2017 and 2022. The study was approved by the Institutional Review Board (Study ID: 2021C0069). Patient data were abstracted from electronic medical records.

Baseline characteristics and renal function were collected on cycle 1, day 1, prior to the administration of the first dose of ICI. All patients received pembrolizumab‐based treatment. Early acute kidney injury (AKI) was defined as any elevation in creatinine levels to ≥ 1.5 times above baseline, occurring from the initiation of pembrolizumab‐based therapy until 21 days after the fourth cycle, or until 21 days after the last cycle for patients who received fewer than 4 cycles. This timeframe was chosen to capture early treatment‐related toxicities, particularly in patients receiving pembrolizumab in combination with chemotherapy; chemotherapy was typically administered during the first 4 cycles [6, 7]. Clinically, this period represented the phase when patients were receiving more intense treatment. The etiologies of AKI were determined by the treating physician at the time of diagnosis and management (Table S1). All AKI cases were adjudicated through a multidisciplinary review involving oncology, hospital medicine, and nephrology to confirm the underlying cause and ensure consistency.

Kaplan–Meier survival analysis and Cox proportional hazards models were used to evaluate and compare overall survival (OS) and hazard ratios (HR) between patients with and without early acute kidney injury. Survival up to 12 months (primary outcome) is measured from the initiation of immunotherapy to either the 12‐month mark or the time of death, whichever occurs first. Baseline characteristics, along with univariate and multivariable survival analysis results, were summarized and presented using forest plots.

As exploratory analyses, Fisher's Exact test was used to examine associations between treatment groups or comorbidities and the development of early AKI. For patients with early AKI, descriptive analyses summarized baseline characteristics, treatment details, and clinical outcomes. To further evaluate clinical outcomes in this subgroup, Kaplan–Meier survival analysis was conducted to compare survival based on maximum creatinine levels (using a cutoff identified with the survminer package in R [29]) and renal recovery status. Additionally, we explored the association between renal recovery and immunotherapy resumption using logistic regression. All statistical analyses were performed using IBM SPSS Statistics (Version 29.0.1.0) and RStudio (Version 2023.12.1).

## 3 Results

**FIGURE 1:**
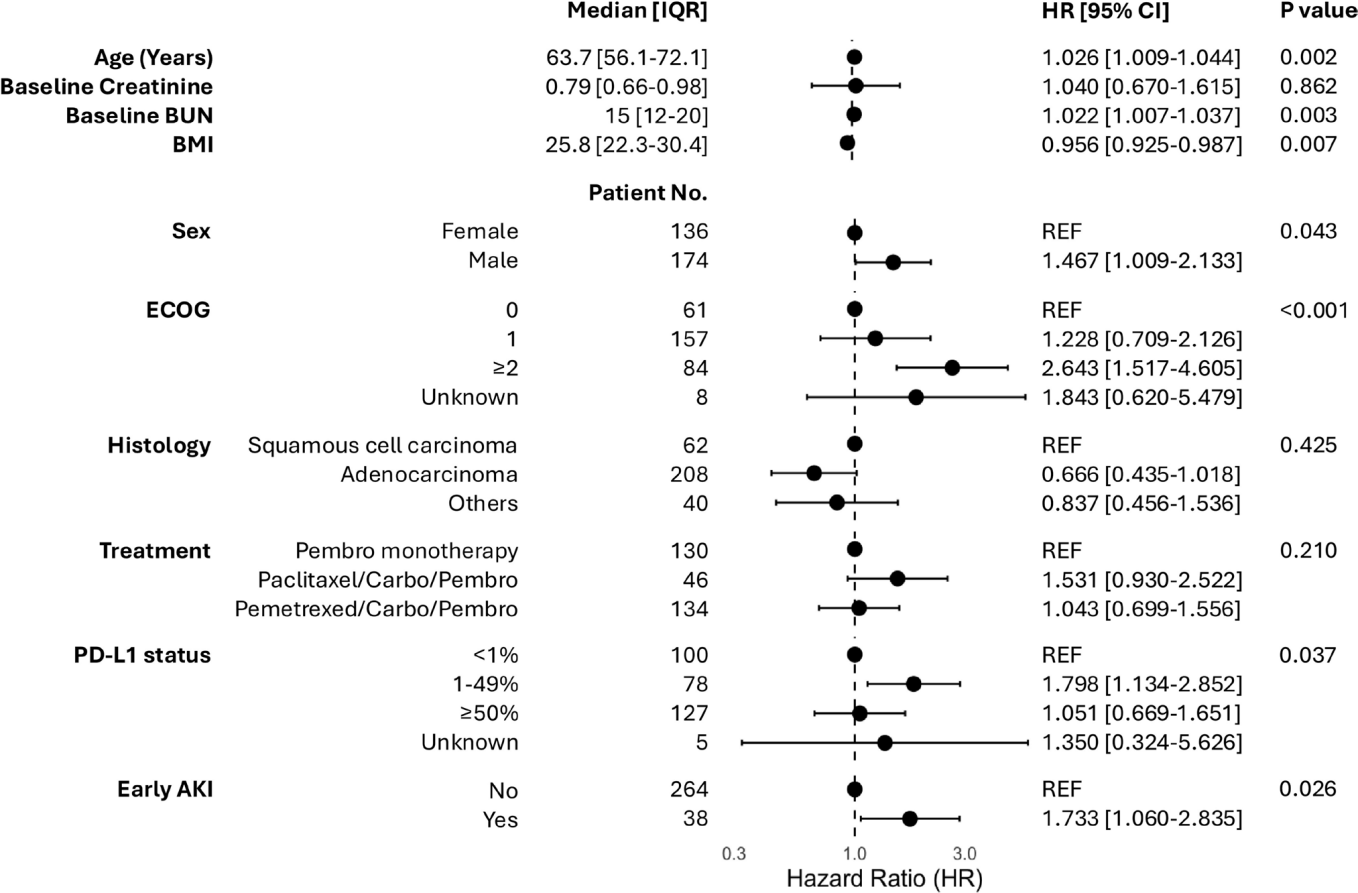
Demographics and corresponding hazard ratio (HR) with 95% confidence interval (CI) and *p*‐values.

A total of 310 patients were included in this study. The median age at the treatment initiation was 63.7 years (interquartile range [IQR], 56.1–72.1). Of these, 174 participants (56.1%) were male, and 136 (43.9%) were female. Most patients (67.1%, *n* = 208) had adenocarcinoma, followed by 62 (20.0%) patients with squamous cell carcinoma, and 40 (12.9%) patients with other histological types. Reflecting standard treatment regimens during the study period, all patients received pembrolizumab‐based immunotherapy, including 134 patients (43.2%) in combination with pemetrexed and carboplatin, 46 patients (14.9%) in combination with paclitaxel and carboplatin, and 130 patients (41.9%) as monotherapy (Figure 1).

Baseline creatinine levels prior to starting immunotherapy‐based treatment were not prognostic for survival within the first 12 months of starting treatment (HR 1.040, 95% CI 0.670–1.615, *p* = 0.862). Conversely, baseline blood urea nitrogen (BUN) levels were a significant prognostic variable, with an HR of 1.022 (95% CI 1.007–1.037, *p* = 0.003). Additional significant predictors of survival up to 12 months included age, body mass index (BMI), sex, Eastern Cooperative Oncology Group (ECOG) performance status, and PD‐L1 status (Figure 1).

Among 310 patients, 302 had repeat creatinine levels measured within the defined period to assess for early AKI. Early AKI developed in 38 patients (12.3%), while 8 patients (2.3%) had unknown AKI status due to lack of repeat creatinine measurements. The median and mean times to AKI development were 42 days (IQR 23–62) and 44.5 days (SD 24.5), respectively.

**FIGURE 2:**
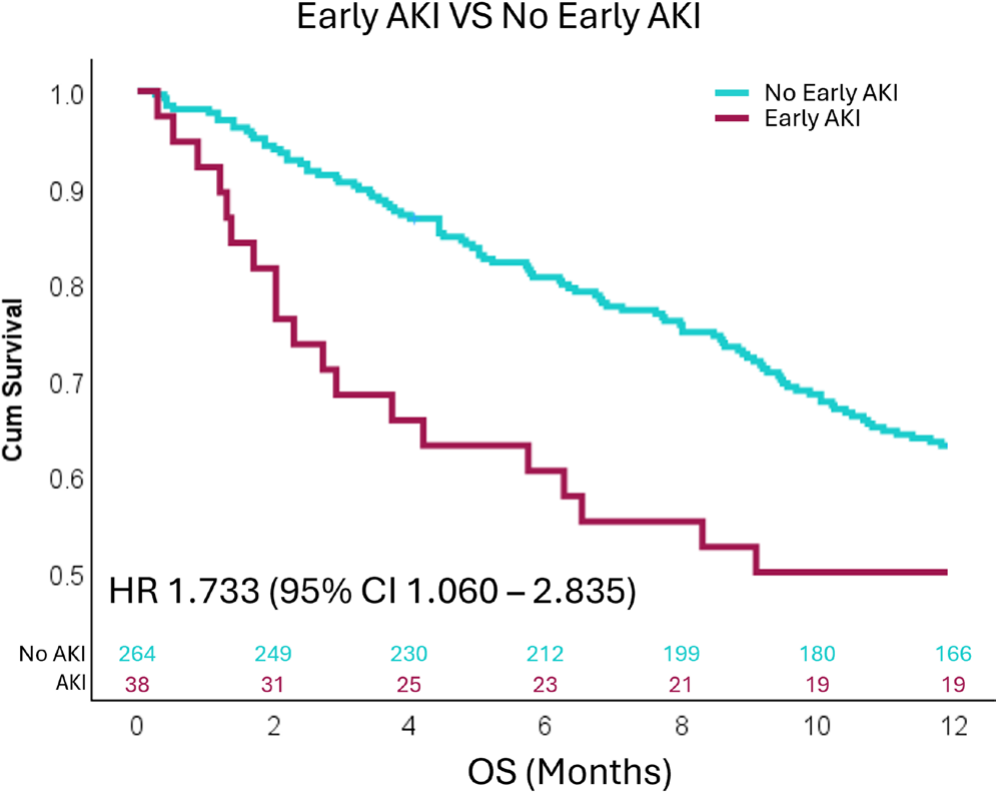
Kaplan–Meier survival analysis showing that patients with early acute kidney injury (AKI) had a significantly higher risk of death within 12 months of initiating front‐line pembrolizumab‐based treatment compared to those without AKI.

**FIGURE 3:**
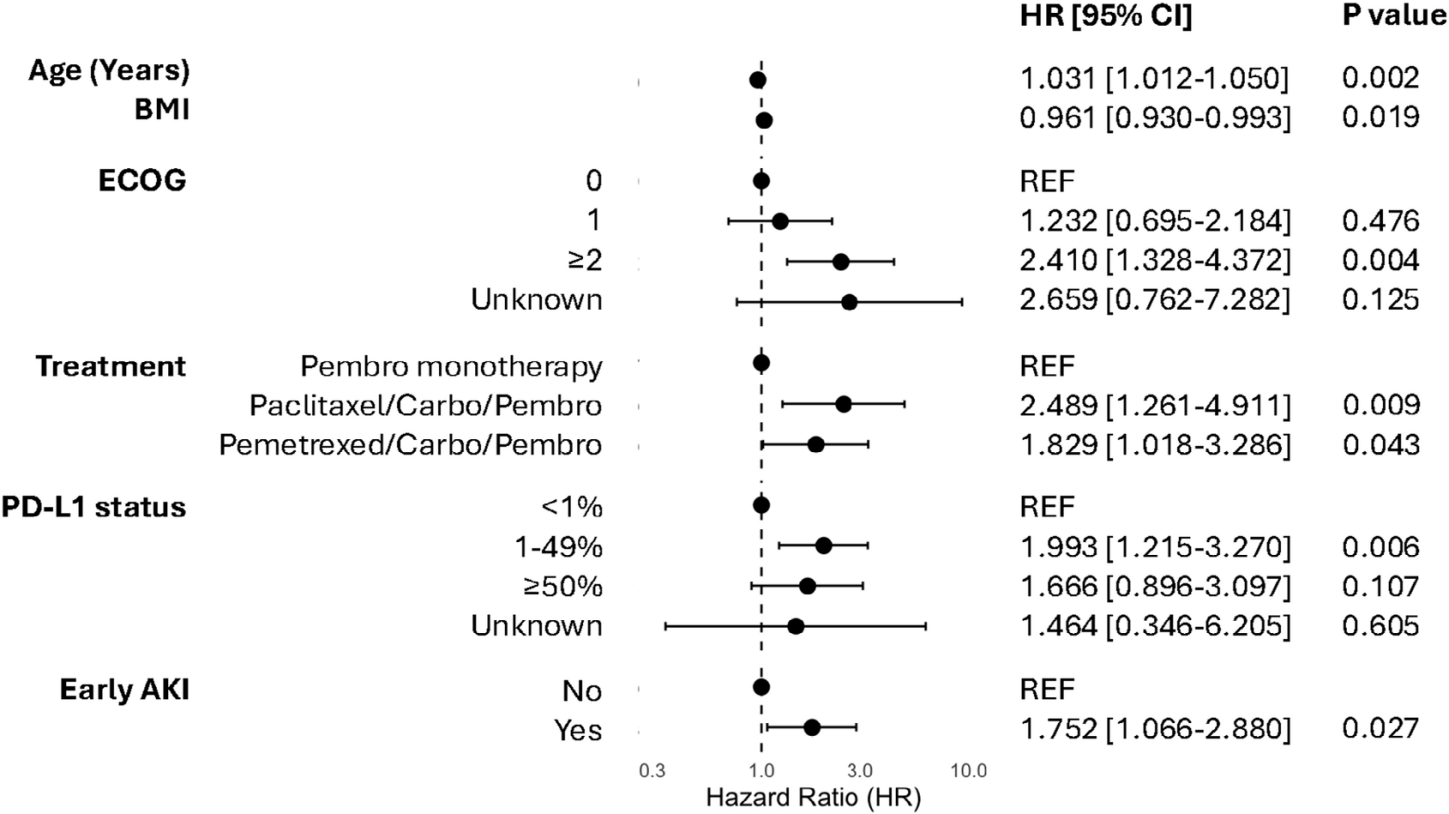
Multivariable analysis showing that patients with early acute kidney injury (AKI) had a significantly higher risk of death, adjusted for body mass index (BMI), age, PD‐L1 expression, ECOG performance status, and treatment type.

The 12‐month survival rate for patients who had developed early AKI was 50.0%, which is significantly lower than those who had not developed early AKI (62.9%). The HR for death within 12 months after starting treatment for patients who had developed early AKI was 1.733 (95% CI 1.060–2.835, *p* = 0.026) (Figure 2). In multivariable analysis after adjusting for age, BMI, ECOG performance status, treatment, PD‐L1 status, and development of AKI remained a statistically significant predictor of reduced12‐month OS (HR: 1.752, 95% CI: 1.066–2.880, *p* = 0.027) (Figure 3).

**FIGURE 4:**
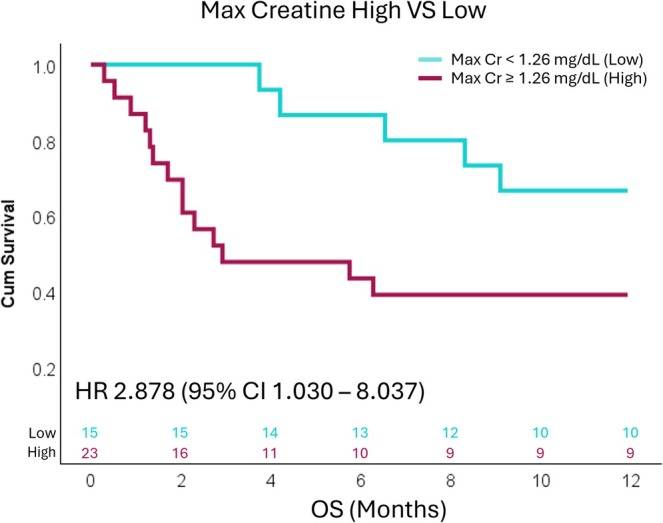
Kaplan–Meier survival analysis demonstrating that patients with early acute kidney injury (AKI) and a maximum creatinine (Cr) level ≥ 1.26 mg/dL (High) had a significantly higher risk of death within 12 months of starting front‐line pembrolizumab‐based treatment compared to those with AKI and a maximum creatinine level < 1.26 mg/dL (Low). The optimal cutoff for maximum creatinine levels to categorize AKI was identified using the survminer package in R.

Among the 38 patients who developed early AKI, the median maximum creatinine level was 1.51 mg/dL (IQR 1.06–2.34), and the median BUN level was 29 mg/dL (IQR 15–61). Decreased 12‐month survival was greater in those with moderately to severely elevated creatinine levels. Subsequently, patients were categorized into high and low maximum creatinine groups, with a cutoff at 1.26 mg/dL. Patients who developed early AKI with creatinine levels ≥ 1.26 mg/dL had a significantly higher risk of death within the first 12 months compared to those with creatinine levels < 1.26 mg/dL, with an HR of 2.878 (95% CI 1.030–8.037, *p* = 0.044) (Figure 4).

**FIGURE 5:**
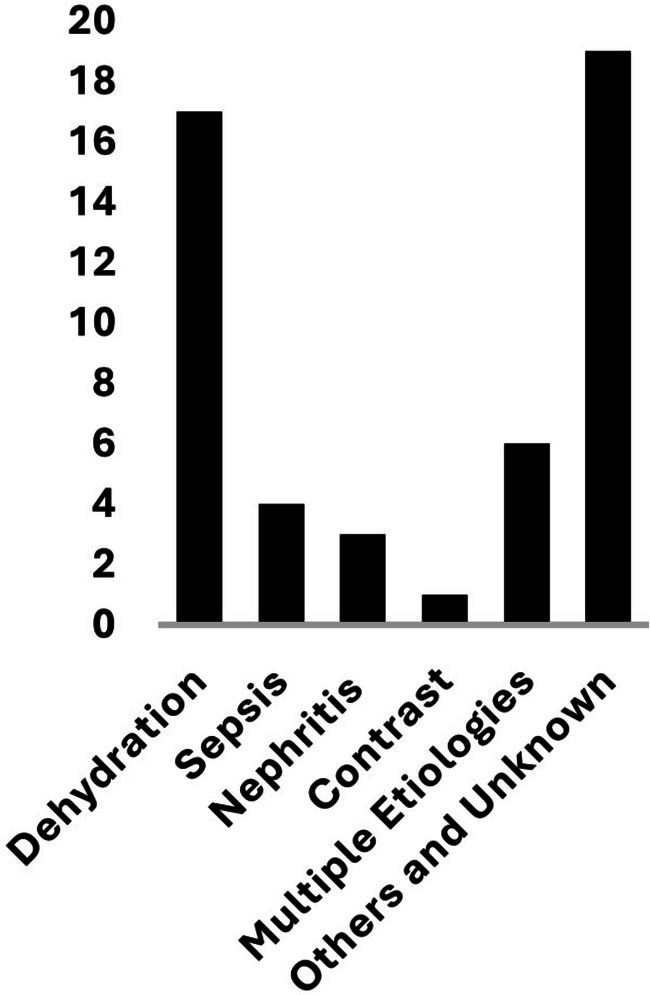
Etiologies of early acute kidney injury (AKI) in patients receiving front‐line pembrolizumab‐based therapy. Among the 38 cases of early AKI, 17 were attributed to dehydration. Four patients developed sepsis‐associated AKI, all of whom also had at least one additional contributing etiology. Three cases were classified as immune checkpoint inhibitor (ICI)‐associated nephritis, including one with concurrent dehydration. One case was related to contrast‐induced nephropathy. Nineteen patients had AKI of unknown etiology or due to causes other than dehydration, sepsis, ICI‐nephritis, or contrast exposure.

The most common etiology of AKI was dehydration, observed in 17 patients (44.7%) who developed AKI. This was followed by sepsis‐associated AKI in 4 patients (10.5%), all of whom had at least one additional contributing factor. ICI‐nephritis was identified in 3 patients (7.9%); among them, one also had concurrent severe dehydration, although ICI‐nephritis remained on the differential diagnosis. Contrast‐induced AKI following computed tomography (CT) imaging occurred in 1 patient (2.6%). In 19 patients (50%), the cause of early AKI was either unknown or contributed to other etiologies. Notably, 6 patients (15.8%) had multiple contributing causes for their early AKI (Figure 5).

Regarding the type of treatment received, patients who received pemetrexed in combination with pembrolizumab and carboplatin had a higher incidence of early AKI compared to those who received paclitaxel with pembrolizumab and carboplatin, with 22 (16.7%) out of 132 patients developing AKI versus 2 (4.5%) out of 44 patients, respectively (Fisher's Exact Test, two tailed *p* = 0.044). In contrast, 14 (11.1%) out of 126 patients who received pembrolizumab monotherapy developed early AKI. The incidence of AKI did not differ significantly between the pembrolizumab monotherapy group and the other treatment groups, as indicated by the Fisher–Freeman–Halton test (two‐tailed *p* = 0.227).

Among the 38 patients who developed AKI, 23 (60.5%) recovered to baseline, and 15 (39.5%) did not recover to baseline. An exploratory analysis was conducted among patients who developed AKI. The 12‐month survival rates were 46.7% for the 15 patients who did not achieve renal recovery and 52.2% for the 23 patients who did. Resuming immunotherapy after early AKI was more common among patients who recovered renal function compared to those who did not, with 14 of 23 (60.9%) resuming treatment versus 4 of 15 (26.7%), respectively. Logistic regression analysis showed that patients who recovered renal function were significantly more likely to resume immunotherapy OR odds ratio (OR) 4.278, 95% CI: 1.036–17.633, *p* = 0.045), indicating an association between renal recovery and treatment resumption. Although patients who resumed treatment after recovery from AKI appeared to have lower odds of death within 12 months (OR = 0.278, 95% CI: 0.048–1.623), the association was not statistically significant (*p* = 0.155).

In this study, 292 patients had available baseline data on medical comorbidities, including diabetes mellitus (DM), hypertension (HTN), and chronic heart failure (CHF). Patients with baseline CHF were significantly more likely to develop early AKI compared to those without CHF (33.3% vs. 11.9%, Fisher's exact test, *p* = 0.032). There was also a trend toward a higher incidence of early AKI among patients with DM (19.4% vs. 11.3%, Fisher's exact test, *p* = 0.134) (Table S2). In multivariate analysis, early AKI remained a statistically significant associated with 12‐month survival after adjusting for comorbidities (Table S3).

## 4 Discussion

The development of AKI during the early course of pembrolizumab‐based front‐line treatment for NSCLC was associated with a significantly increased risk of death within the first 12 months of therapy. In our cohort, 12.6% of patients experienced early AKI, and the negative association with survival remained significant after adjusting for key factors such as age, treatment regimen, tumor PD‐L1 expression, and clinical performance status. Our findings highlight the need to better understand the biological mechanism for this association, as well as the need for vigilant renal function monitoring and optimization in patients during the early course of treatment.

The etiologies of AKI were diverse in this study, including dehydration, CT contrast exposure, sepsis, and nephritis, consistent with prior reports [29, 30]. Moderate to severe AKI was associated with significantly reduced survival within the first 12 months of starting immunotherapy, while mild AKI showed outcomes similar to those without AKI. Given dehydration was the most common cause, it often related to gastrointestinal side effects such as nausea, vomiting, and poor oral intake [31, 32]. These findings underscore the need for closer symptom and weight monitoring, even between routine oncology visits. Home‐based tools for symptom tracking and weight measurement may help detect early signs of dehydration and reduce the risk of severe AKI [33, 34]. Future studies are needed to evaluate the impact of such interventions on patient outcomes.

Among the contributing factors to AKI, treatment‐specific agents play a significant role. Pemetrexed, a commonly used agent, is known to cause AKI [35, 36]. As expected, patients who received pemetrexed in combination with pembrolizumab and carboplatin had significantly higher rates of early AKI compared to those receiving paclitaxel‐based treatment, emphasizing the need for special attention in this subgroup. Surprisingly, patients receiving pembrolizumab monotherapy, a regimen traditionally considered less nephrotoxic, had a numerically higher rate of AKI compared to those on paclitaxel‐based therapy, with rates comparable to those in the pemetrexed‐based group [37]. We hypothesize that several factors may contribute to the lower observed incidence of AKI in the combination chemotherapy group with paclitaxel, including the routine use of corticosteroids, increased administration of intravenous fluids, and the likelihood that these patients are more symptomatic, prompting earlier medical interventions that may have mitigated the development of AKI. Future studies are needed to further confirm and investigate the unexpectedly high AKI rates associated with pembrolizumab monotherapy.

Although a substantial proportion of patients recovered from AKI, their overall 12‐month survival was comparable to those who did not recover. In exploratory analysis, patients who were able to resume immunotherapy demonstrated a numerically higher 12‐month survival rate compared to those who were not, though this difference did not reach statistical significance. These nonsignificant results may be due to the limited sample size, and caution is warranted when interpreting these exploratory analyses. However, this may also reflect unmeasured clinical factors associated with AKI that were not captured by available variables. Emerging evidence suggests that renal inflammation following AKI could potentially alter the expression of the neonatal Fc receptor (FcRn) on podocytes and promote transporting IgG from the glomerular basement membrane into the urinary space, increasing renal IgG clearance [38, 39]. Accelerated ICI clearance, in turn, has been associated with poor clinical outcomes [40]. Additionally, treating AKI in the setting of sepsis with antibiotics may alter the microbiome, which has been shown to dampen ICI effectiveness by down‐regulating mucosal addressin cell adhesion molecule 1 (MAdCAM‐1) [41, 42]. Further research is warranted to better understand the factors driving poor outcomes in this group.

While our study demonstrates a significant association between early AKI development and shorter survival within the first 12 months of pembrolizumab‐based treatment, several limitations should be acknowledged. Due to the retrospective nature of our analysis, no causal relationships can be established. Additionally, the small size of the AKI cohort in combination with the diverse etiologies of AKI limited our ability to conduct more detailed exploratory subgroup analyses evaluating the relationship between specific causes and risk factors of AKI and clinical outcomes. Furthermore, because laboratory data were typically obtained every 3 weeks in the outpatient setting for the majority of patients, we were unable to capture short‐term changes in creatinine or urine output, preventing the application of standardized AKI classification systems such as KDIGO or AKIN for patients who did not require hospitalization. As such, our definition of AKI was based solely on changes in serum creatinine, which, while pragmatic in clinical practice, may lack the granularity of validated staging systems. Lastly, our study exclusively included patients receiving pembrolizumab‐based therapies, and the incidence, timing, and outcomes of AKI may differ with other FDA‐approved immunotherapy regimens, such as dual checkpoint blockade oranti‐PD‐L1 monoclonal antibodies.

## 5 Conclusion

The development of early AKI during the initial phase of pembrolizumab‐based treatment in patients with metastatic NSCLC was associated with reduced 12‐month overall survival. These findings highlight the critical need for vigilant monitoring and the adoption of preventive measures to mitigate AKI risk. Further research is warranted to elucidate the mechanisms driving AKI development and to refine management strategies for patients receiving immunotherapy.

## Author Contributions

**Mingjia Li:** conceptualization (lead), data curation (lead), formal analysis (equal), funding acquisition (supporting), investigation (lead), methodology (lead), project administration (lead), resources (supporting), supervision (lead), validation (equal), visualization (lead), writing – original draft (lead), writing – review and editing (lead). **Po‐Lan Su:** formal analysis (equal), investigation (supporting), methodology (supporting), validation (equal), visualization (supporting), writing – original draft (supporting), writing – review and editing (supporting). **Songzhu Zhao:** formal analysis (equal), investigation (supporting), methodology (supporting), validation (equal), visualization (supporting), writing – review and editing (supporting). **Grace Cheng:** data curation (supporting), writing – original draft (supporting), writing – review and editing (supporting). **Nicholas Jones:** data curation (supporting), investigation (supporting), writing – review and editing (supporting). **Kenneth Chian:** data curation (supporting), investigation (supporting), writing – review and editing (supporting). **Natalie Johns:** data curation (supporting), investigation (supporting), writing – review and editing (supporting). **Adam Khorasanchi:** data curation (supporting), investigation (supporting), writing – review and editing (supporting). **Adeoluwa Adeluola:** investigation (supporting), writing – original draft (supporting), writing – review and editing (supporting). **Annastasia Petouhoff:** investigation (supporting), visualization (supporting), writing – review and editing (supporting). **Daniel J. Spakowicz:** investigation (supporting), visualization (supporting), writing – review and editing (supporting). **Lai Wei:** formal analysis (equal), investigation (supporting), methodology (supporting), validation (supporting), visualization (supporting), writing – review and editing (supporting). **Xiaoying Wang:** investigation (supporting), writing – review and editing (supporting). **Christopher Coss:** investigation (supporting), writing – review and editing (supporting). **Mitch A. Phelps:** conceptualization (supporting), investigation (supporting), writing – review and editing (supporting). **Lingbin Meng:** investigation (supporting), writing – review and editing (supporting). **Yuanquan Yang:** investigation (supporting), writing – review and editing (supporting). **Alexa Meara:** conceptualization (supporting), investigation (supporting), investigation (supporting), writing – review and editing (supporting), writing – review and editing (supporting). **Mohamed I. Elsaid:** investigation (supporting), writing – review and editing (supporting). **Jinesh Gheeya:** investigation (supporting), writing – review and editing (supporting). **Logan Roof:** data curation (supporting), investigation (supporting), writing – review and editing (supporting). **Asrar Alahmadi:** data curation (supporting), investigation (supporting), writing – review and editing (supporting). **Regan M. Memmott:** data curation (supporting), investigation (supporting), writing – review and editing (supporting). **Jacob Kaufman:** data curation (supporting), investigation (supporting), writing – review and editing (supporting). **Kai He:** data curation (supporting), investigation (supporting), writing – review and editing (supporting). **Carolyn J. Presley:** data curation (supporting), investigation (supporting), writing – review and editing (supporting). **Peter G. Shields:** data curation (supporting), investigation (supporting), writing – review and editing (supporting). **David P. Carbone:** data curation (supporting), investigation (supporting), writing – review and editing (supporting). **Gregory A. Otterson:** conceptualization (supporting), data curation (supporting), investigation (supporting), writing – review and editing (supporting). **Jason Prosek:** conceptualization (supporting), investigation (supporting), writing – original draft (supporting), writing – review and editing (supporting). **Dwight H. Owen:** conceptualization (supporting), data curation (supporting), formal analysis (supporting), funding acquisition (lead), investigation (supporting), methodology (supporting), project administration (equal), resources (lead), supervision (supporting), validation (supporting), visualization (supporting), writing – original draft (supporting), writing – review and editing (supporting).

## Ethics Statement

Institutional Review Board Approval: This study was approved by the Institutional Review Board (IRB) at the Ohio State University (Study ID: 2021C0069).

## Conflicts of Interest

Christopher Coss Patents: Patent holder concerning IP discovered while employed by GTx INC. Patent holder and royalty recipient from Recursion Pharmaceuticals concerning IP surrounding methods of use for HDACi AR‐42. He Kai. Consulting or Advisory Role: Perthera, Mirati Therapeutics, Bristol‐Myers Squibb, Iovance Biotherapeutics, Geneplus, Lyell Immunopharma, and AstraZeneca. Research Funding: Bristol‐Myers Squibb, Mirati Therapeutics, Adaptimmune, Genentech/Roche, GlaxoSmithKline, Amgen, Abbvie, Oncoc4. David P. Carbone. Consulting or Advisory Role: Merck, AstraZeneca, Bristol‐Myers Squibb, EMD Serono, GlaxoSmithKline, Janssen, Genentech/Roche, Intellisphere, Lilly, Mirati Therapeutics, Johnson & Johnson/Janssen, Sanofi, Abbvie, Regeneron, PPD, Curio Science, Iovance Biotherapeutics, Jazz Pharmaceuticals, Merck KGaA, Novartis, Roche, InThought, OncLive/MJH Life Sciences, Pfizer, Arcus Biosciences, NCCN/AstraZeneca, MSD Oncology. Honoraria: AstraZeneca, Bristol‐Myers Squibb/Ono Pharmaceutical. Gregory A. Otterson. Consult or Advisory Role: Novocure, OncLive/MJH Life Sciences. Research Funding: Genentech/Roche, Pfizer, Bristol‐Myers Squibb, Novartis, Merck, AstraZeneca, Revolution Medicines, Array BioPharma, Apollomics. Carolyn J. Presley. Consulting or Advisory Role: OncLive, Regeneron. Dwight H. Owen. Travel, Accommodation, Expenses: Genentech. Research Funding: Bristol‐Myers Squibb, Palobiofarma, Merck Sharp & Dohme, Genentech/Roche, Onc.AI.

## Supporting information


**Table S1** Etiologies of acute AKI were classified as dehydration, sepsis‐associated, immune‐related nephritis (ICI‐nephritis), contrast‐induced nephropathy, or unknown.
**Table S2**. Comparison of comorbidities between patients with and without early AKI.
**Table S3**. Multivariable survival analysis additionally adjusted for comorbidities. Early AKI remained a significantly associated with 12‐month survival after adjusting for Eastern Cooperative Oncology Group performance status (ECOG), treatment, age, body mass index (BMI), PD‐L1 expression levels (negative < 1%, low 1%–49%, high ≥ 50%), diabetes mellitus (DM), hypertension (HTN), and chronic heart failure (CHF).

## Data Availability

The data supporting the findings of this study are available upon request from the corresponding author and are subject to approval by The Ohio State University. The data is not publicly available due to privacy restrictions.
