# Supplementary material for: Early Acute Kidney Injury and Its Association With Survival in Patients With Metastatic Non‐Small‐Cell Lung Cancer Treated With Front‐Line Immunotherapy‐Based Therapies
Source: Cancer Med. 2025 Jul 28;14(15):e71058. doi: 10.1002/cam4.71058 (PMC12302575; doi:10.1002/cam4.71058)
Supplement: Supplementary file 1 — Table S1 Etiologies of acute AKI were classified as dehydration, sepsis‐associated, immune‐related nephritis (ICI‐nephritis), contrast‐induced nephropathy, or unknown. Table S2. Comparison of comorbidities between patients with and without early AKI. Table S3. Multivariable survival analysis additionally adjusted for comorbidities. Early AKI remained a significantly associated with 12‐month survival after adjusting for Eastern Cooperative Oncology Group performance status (ECOG), treatment, age, body mass index (BMI), PD‐L1 expression levels (negative < 1%, low 1%–49%, high ≥ 50%), diabetes mellitus (DM), hypertension (HTN), and chronic heart failure (CHF). [file CAM4-14-e71058-s001.docx]

Supplementary Methods:

Etiologies of acute AKI and definitions

| Etiology | Definition |
| --- | --- |
| Dehydration | AKI occurring with clinical evidence of low oral intake and/or significant fluid losses (e.g., vomiting, diarrhea). |
| Sepsis-associated | Decline in renal function in the presence of a clinically or microbiologically confirmed infection. |
| CT Contrast-induced | AKI occurring shortly after intravenous contrast exposure, with no other identifiable cause. |
| ICI-nephritis | AKI occurring after immune checkpoint inhibitor (ICI) exposure, with no alternative identifiable cause (e.g., infection, dehydration, or recent contrast), supported by urinalysis and biopsy if available, as assessed by nephrologist(s). |
| Other etiologies or unknown | Unknown or contributed to other etiologies other than dehydration, sepsis-associated, contrast-induced, ICI-nephritis |

Table S1. The etiologies of acute AKI were classified as dehydration, sepsis-associated, immune-related nephritis (ICI-nephritis), contrast-induced nephropathy, or unknown.

Results:

Out of 310 patients, 292 patients had baseline medical comorbidities reported.

| **Variable** | **Level** | **No Early AKI (n=264)** | **Early AKI (n=38)** | **Total (n=302)** | **p-value** |
| --- | --- | --- | --- | --- | --- |
| Diabetes | Missing | 10 | 0 | 10 | 0.134 |
|  | No | 204 (88.7%) | 26 (11.3%) | 230 (78.8%) |  |
|  | Yes | 50 (80.7%) | 12 (19.3%) | 62 (21.2%) |  |
| Hypertension | Missing | 10 | 0 | 10 | 0.862 |
|  | No | 113 (87.6%) | 16 (12.4%) | 129 (44.2%) |  |
|  | Yes | 141 (86.5%) | 22 (13.5%) | 163 (55.8%) |  |
| Heart Failure | Missing | 10 | 0 | 10 | 0.032 |
|  | No | 244 (88.1%) | 33 (11.9%) | 277 (94.9%) |  |
|  | Yes | 10 (66.7%) | 5 (33.3%) | 15 (5.1%) |  |

Table S2. Comparison of comorbidities between patients with and without early AKI.

| **Variable** | **Comparison** | **HR** | **95% CI** | | **p-value** |
| --- | --- | --- | --- | --- | --- |
| Early AKI | Yes vs No | 1.748 | 1.057 | 2.891 | 0.030 |
| ECOG | 1 vs 0 | 1.151 | 0.642 | 2.064 | 0.636 |
|  | ≥2 vs 0 | 2.406 | 1.311 | 4.417 | 0.005 |
| Treatment | Paclitaxel/Carbo/Pembro vs Pembro | 2.482 | 1.233 | 4.998 | 0.011 |
|  | Pemetrexed/Carbo/Pembro vs Pembro | 1.994 | 1.091 | 3.647 | 0.025 |
| Age |  | 1.027 | 1.007 | 1.048 | 0.009 |
| BMI |  | 0.958 | 0.924 | 0.993 | 0.020 |
| PD-L1 | Low vs Negative | 2.155 | 1.291 | 3.598 | 0.003 |
|  | High vs Low | 1.721 | 0.909 | 3.259 | 0.096 |
| DM | Yes vs No | 1.371 | 0.838 | 2.244 | 0.209 |
| HTN | Yes vs No | 0.872 | 0.579 | 1.314 | 0.513 |
| CHF | Yes vs No | 0.930 | 0.389 | 2.224 | 0.871 |

Table S3. Multivariable survival analysis additionally adjusted for comorbidities. Early AKI remained a significantly associated with 12-month survival after adjusting for Eastern Cooperative Oncology Group performance status (ECOG), treatment, age, body mass index (BMI), PD-L1 expression levels (negative <1%, low 1-49%, high ≥50%), diabetes mellitus (DM), hypertension (HTN), and chronic heart failure (CHF).
